# Pain and gain of predictive genetic testing: Particular case of fragile X syndrome

**DOI:** 10.1192/j.eurpsy.2024.1221

**Published:** 2024-08-27

**Authors:** N. Bouayed Abdelmoula, N. Ramma, B. Abdelmoula

**Affiliations:** Genomics of Signalopathies at the service of Precision Medicine - LR23ES07, Medical University of Sfax, Sfax, Tunisia

## Abstract

**Introduction:**

The purpose of predictive genetic tests is to identify carriers or the onset of a disease in pre-symptomatic individuals. Prediction is linked to a negative psychological impact (anxiety, depression, etc.), depending on the perception of risk, the severity of the disease, and the availability and effectiveness of treatments.

**Objectives:**

Here, we report on genetic counselling during predictive genetic testing offered to an Arab family affected by fragile X condition (FXS) caused by the unstable expansion of a CGG repeat (CGGR) in the FMR1 gene.

**Methods:**

A 10-year-old boy who harbored a mental retardation was referred to our genetic counselling for genetic testing as he was suspected to be affected by FXS. Screening of FMR1 gene mutations was conducted for the index case and his mother. A predictive genetic testing for the family members (brothers, sisters and others) was offered, focusing on knowledge of genetics and medical risks of FXS.

**Results:**

FMR1 molecular analysis showed a full mutation (300 to 2000 CGGR) for the boy and a large premutation (100 CGGR) for the mother. During genetic counselling, the family was informed about the significance of the genetic results. In FXS initiated by an expansion of over 200 CGGR. While mental retarded males usually harbor the full mutation, the mother carry a premutation (70 to 200 CGGR). The deficiency of FMR protein (FMRP) in the neurons of affected males leads to brain developmental abnormalities. Some pre-mutated children may show signs of the autism spectrum disorder and females may develop FMR1-related premature ovarian insufficiency. An increased risk of a late onset fragile X tremor ataxia syndrome is identified in pre-mutated men (55 to 200 CGGR) and less in women.

**Conclusions:**

The reduction or loss of FMRP leads to multisystem damage. Neuropsychiatric disorders such as mental retardation, speech and language delay, autism spectrum disorder, sensory hyperexcitation, social anxiety, abnormal eye contact, shyness and aggressive behaviour are common in individuals with the mutation. Affected women are often under-diagnosed because mental retardation is not constant, but minor disorders including a borderline IQ with learning difficulties and emotional disturbances have been reported. Conditions associated with fragile X premutation, a term proposed by the European Fragile X Network (FXPAC), seem to be characterized by many physical and psychological health symptoms. Anxiety, depression, sleep disorders and mood disorders are more common in permutated individuals. However, new reports suggested that FXS patients could be at unusually low risk of cancers, because FMRP is over-expressed in multiple cancer tissues.

**Disclosure of Interest:**

None Declared

